# Hybrid Collision Avoidance for ASVs Compliant With COLREGs Rules 8 and 13–17

**DOI:** 10.3389/frobt.2020.00011

**Published:** 2020-02-11

**Authors:** Bjørn-Olav H. Eriksen, Glenn Bitar, Morten Breivik, Anastasios M. Lekkas

**Affiliations:** Department of Engineering Cybernetics, Centre for Autonomous Marine Operations and Systems, Norwegian University of Science and Technology, Trondheim, Norway

**Keywords:** hybrid collision avoidance, autonomous surface vehicle (ASV), COLREGs, COLREGs compliant, model predictive control (MPC), energy-optimized control

## Abstract

This paper presents a three-layered hybrid collision avoidance (COLAV) system for autonomous surface vehicles, compliant with rules 8 and 13–17 of the International Regulations for Preventing Collisions at Sea (COLREGs). The COLAV system consists of a high-level planner producing an energy-optimized trajectory, a model-predictive-control-based mid-level COLAV algorithm considering moving obstacles and the COLREGs, and the branching-course model predictive control algorithm for short-term COLAV handling emergency situations in accordance with the COLREGs. Previously developed algorithms by the authors are used for the high-level planner and short-term COLAV, while we in this paper further develop the mid-level algorithm to make it comply with COLREGs rules 13–17. This includes developing a state machine for classifying obstacle vessels using a combination of the geometrical situation, the distance and time to the closest point of approach (CPA) and a new CPA-like measure. The performance of the hybrid COLAV system is tested through numerical simulations for three scenarios representing a range of different challenges, including multi-obstacle situations with multiple simultaneously active COLREGs rules, and also obstacles ignoring the COLREGs. The COLAV system avoids collision in all the scenarios, and follows the energy-optimized trajectory when the obstacles do not interfere with it.

## 1. Introduction

Motivated by the potential for reduced costs and increased safety, the maritime industry is rapidly moving toward autonomous operations. Following groundbreaking advances in the automotive industry, many sectors within the maritime industry are considering the benefits of autonomy, which includes more environmentally friendly operations. For instance, the agricultural chemical company *Yara* together with the maritime technology supplier *Kongsberg Maritime* are developing the electrical autonomous container vessel *Yara Birkeland*, which aims to replace 40,000 yearly truck journeys in urban eastern Norway^1^. Another example is the world's first autonomous car ferry, *Falco*, developed by *Rolls-Royce* (recently bought by Kongsberg Maritime) and *Finferries*. In 2018, *Falco* navigated autonomously between two ports in Finland^2^. Reports state that in excess of 75 % of maritime accidents are due to human errors (Chauvin, 2011; Levander, 2017), indicating that there is also a potential for increased safety in addition to the economical and environmental benefits.

An obvious prerequisite for autonomous ship operations is the development of robust and well-functioning COLAV systems. In addition to generating collision-free maneuvers, a COLAV system must adhere to the “rules of the road” of the oceans, i.e., the COLREGs (Cockcroft and Lameijer, 2004). These rules are written for human ship operators and include qualitative requirements on how to perform safe and readily observable maneuvers. Part B of the COLREGs concern steering and sailing, and includes the following rules that are the most relevant to a motion control system:

**Table:** 

**Rule 8**	Requires maneuvers to be readily observable and to be done in ample time.
**Rules 13–15**	Describe the maneuvers to perform in cases of overtaking, head-on and crossing situations. Participants in crossing situations are defined by the terms *give-way* and *stand-on* vessels.
**Rule 16**	Requires that a give-way vessel must take early and substantial action to keep clear of the stand-on vessel.
**Rule 17**	Consists of two main parts. The first part requires a stand-on vessel to maintain its course and speed, while the second part allows/requires^3^ a stand-on vessel to take action to avoid collision if the give-way vessel is not taking action.

Since the rules are written for humans, with few quantitative figures, a challenge for autonomous operation is to quantify them into behaviors that can be executed algorithmically. The focus of the work in this paper is to do that, and to design a hybrid COLAV system that performs motion planning and generates maneuvers in compliance to rules 8 and 13–17 of the COLREGs.

A number of COLAV approaches considering the COLREGs have been proposed in the past. This includes algorithms using simulation-based model predictive control (Hagen et al., 2018), velocity obstacles (Kuwata et al., 2014), rule-based repairing A* (Campbell et al., 2014), and interval programming (Benjamin et al., 2006). All these approaches are single-layer approaches, where one algorithm solves the complete COLAV problem.

Another approach to the COLAV problem is to use a hybrid architecture, where the task of planning an obstacle-free path or trajectory, complying with the COLREGs and ultimately performing safe maneuvers is divided into layers in a control hierarchy. The idea of hybrid architectures is to divide the subtasks of the COLAV problem into multiple algorithms, exploiting their complementary strengths. This also has the side effect of making it easier for human operators or supervisors to understand the system. Most single-layer algorithms use sample-based approaches that consider a finite number of discrete control inputs, as opposed to conventional gradient-based search algorithms. The reason for this is that many gradient-based algorithms are not sufficiently numerically robust, not allowing a COLAV system to solely rely on such an algorithm. This issue can be handled in hybrid architectures, constrained by the bottom-level algorithm being numerically robust and able to handle extraordinary situations where the other algorithms fail. Hence, hybrid architectures also allows using gradient-based algorithms, which are able to solve problems with large search spaces more efficiently than sample-based algorithms. The works by Loe (2008) and Švec et al. (2013) are examples of two-layered hybrid COLAV architectures. The top layers perform trajectory planning among static obstacles, while the bottom layers perform moving obstacle avoidance in compliance with COLREGs rules 13–16. Casalino et al. (2009) presents a three-layered hybrid COLAV system where the top layer also performs trajectory planning amongst static obstacles. The middle layer avoids moving obstacles, while the bottom layer implements safety functions for handling cases where the two other layers fail. This approach does, however, not consider the COLREGs.

**Figure 1:**
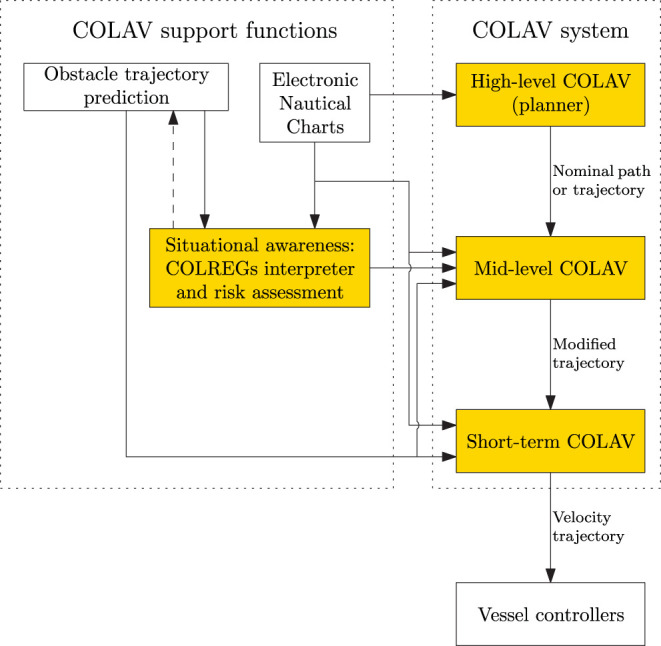
Hybrid architecture with three COLAV layers, where the highlighted functions mark the areas of interest in this article. The COLAV system consists of a high-level planner, a mid-level COLAV algorithm and a short-term COLAV algorithm. The COLAV system is supported by data from electronic nautical charts, represented in a suitable manner for the algorithms, as well as situational awareness functions that track and predict obstacles, interpret the COLREGs and perform risk assessment.

Figure 1 shows a three-layered hybrid COLAV system for an autonomous surface vehicle (ASV). The authors have previously worked extensively on different components of this architecture. Examples include high-level COLAV algorithms (Bitar et al., 2018, 2019b), a mid-level algorithm (Eriksen and Breivik, 2017b; Bitar et al., 2019a), short-term algorithms (Eriksen et al., 2018, 2019; Eriksen and Breivik, 2019) and the development of high-performance vessel controllers (Eriksen and Breivik, 2017a, 2018).

In this paper, we demonstrate the three-layered hybrid COLAV shown in Figure 1 by combining and extending the COLAV algorithms developed in Eriksen and Breivik (2017b, 2019); Bitar et al. (2019a,b); Eriksen et al. (2019). The high-level planner has a long temporal horizon, and finds an energy-optimized nominal trajectory from an initial to a goal position. It considers static obstacles, which may include bathymetric constraints. Since the high-level planner only considers static information, it is intended to be run offline, but it can also be run online, for instance if new static obstacles are detected. The mid-level algorithm attempts to follow the nominal trajectory, while performing COLAV of static and moving obstacles in compliance with COLREGs rules 8, 13–16, and the first part of Rule 17. The mid-level algorithm is run periodically with a shorter temporal horizon than the high-level algorithm, and produces a modified trajectory which is passed to the short-term layer. Both the high-level and mid-level algorithms use gradient-based optimization. The short-term algorithm attempts to follow the modified trajectory, while it in compliance with the second part of Rule 17 handles situations where obstacles ignore the COLREGs. This algorithm also handles other emergency situations, and uses sample-based optimization to achieve a high level of robustness, ensuring safe operation if the mid-level algorithm fails to find a solution. The following list summarizes our contributions:

- The high-level planner from Bitar et al. (2019b) is modified to include the mathematical model of the *Telemetron* ASV in Bitar et al. (2019a), including ocean currents.
- The development of a state-machine-based COLREGs interpretation scheme.
- The mid-level COLAV from Bitar et al. (2019a) is modified to include rules 13–16 and the first part of Rule 17.
- The branching-course model predictive control (BC-MPC) algorithm for short-term COLAV is modified to reduce oscillatory behavior in turns.
- The three-layered COLAV system is verified in simulations and shown to be compliant with rules 8 and 13–17.

The rest of the paper has the following structure: The mathematical model of the ASV *Telemetron* is described in section 2. The high-level planner, mid-level and short-term COLAV algorithms are described in sections 3–5, respectively. In section 6 we present and discuss the simulation scenarios and results, and we conclude the paper in section 7.

## 2. ASV Modeling

**Figure 2:**
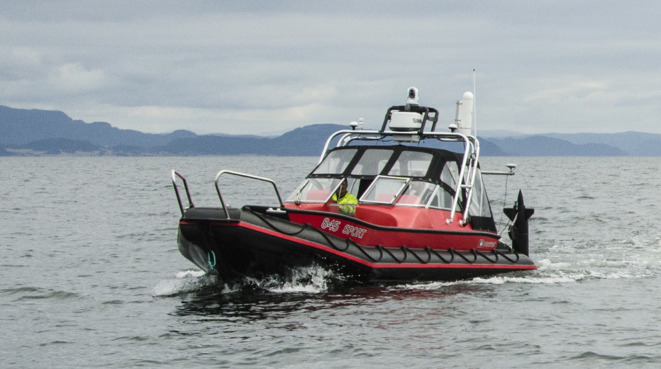
The Telemetron ASV, designed for both manned and unmanned operations. Courtesy of Maritime Robotics.

The vessel of interest in this article is the Telemetron ASV, which is owned and operated by the Norwegian company Maritime Robotics and shown in Figure 2. The Telemetron ASV is a high-speed dual-use vessel propelled by a steerable outboard engine, capable of speeds up to 18 m/s.

Eriksen and Breivik (2017a) presents a model of the Telemetron ASV, which is extended to include ocean currents in Bitar et al. (2019a). The model has the form

(1) $$\begin{array}{l} \dot{\eta }=\text{R}(\psi ){\text{x}}_{r}+{\left[\begin{array}{cc} {\text{V}}_{c}^{⊤} & 0 \end{array}\right]}^{⊤}\\ \;\text{M}({\text{x}}_{r}){\dot{\text{x}}}_{r}+\sigma (x_{r})=\tau , \end{array}$$

where **η** = [*x, y*, ψ]^⊤^ ∈ ℝ^2^ × *S* is the vessel pose and ${\text{V}}_{c}={\left[V_{x},V_{y}\right]}^{⊤}$ describes the ocean current, both in the Earth-fixed North-East-Down frame {*n*}. The vector ${\text{x}}_{r}={\left[u_{r},r\right]}^{⊤}\in {𝕏}_{r}\subset {\mathbb{R}}^{2}$ is the vessel velocity under the assumption of zero relative sway motion (Bitar et al., 2019a), where the set 𝕏_*r*_ describes the vessel-feasible steady-state velocities where (1) is valid. The transformation matrix ***R***(ψ) is given by the heading ψ ∈ *S* as

(2) $$\text{R}(\psi )=\left[\begin{array}{cc} \text{cos }\psi & 0\\ \text{sin }\psi & 0\\ 0 & 1 \end{array}\right],$$

while *r* ∈ ℝ describes the vessel yaw-rate. The matrix ***M***(***x***_*r*_) is a state-dependent inertia matrix, while **σ**(***x***_*r*_) and $\tau ={\left[\tau_{m},\tau_{\delta }\right]}^{⊤}\in 𝕌\subset {\mathbb{R}}^{2}$ describe the vessel damping and control input, respectively. The set 𝕌 describes the control inputs where (1) is valid.

In this work, we assume that the ocean current ***V***_*c*_ is constant and known. For practical applications, the ocean current can be measured via appropriate instrumentation, estimated via sensor fusion methods, or predicted based on e.g., tide tables or sensor networks, such as the European marine observation and data network^4^.

## 3. High-Level Planner

To plan the ASV's nominal trajectory, we use a high-level trajectory planner developed in Bitar et al. (2019b). This trajectory planner uses the ASV model described in section 2 to generate an energy-optimized trajectory between the start and goal positions. The planning algorithm combines an A^⋆^ implementation and an optimal control problem (OCP) solver to generate a feasible and optimized trajectory.

The high-level planning algorithm consists of three steps: First the A^⋆^ implementation finds the shortest piecewise linear path between the start and goal position. Secondly, artificial temporal information is added to the path, converting it to a trajectory of states and inputs. Finally, the trajectory is used as an initial guess for an OCP solver, which finds a locally energy-optimized trajectory near the shortest path. All steps account for static obstacles in the form of elliptical boundaries.

### 3.1. Static Obstacles

The elliptical boundaries are described with the inequality:

(3) $${\left(\frac{x-x_{c}}{x_{a}}\right)}^{2}+{\left(\frac{y-y_{c}}{y_{a}}\right)}^{2}\geq 1,$$

where *x*_*c*_ and *y*_*c*_ is the ellipsis center, and *x*_*a*_, *y*_*a*_ > 0 are the ellipsis major and minor axes, respectively. To allow for angled obstacles, the ellipses are rotated clockwise by an angle α. We add a small constant ϵ > 0 to each side of the inequality, and take the logarithm to arrive at the following obstacle representation:

(4) $$\begin{array}{l} h_{o}(x,y,x_{c},y_{c},x_{a},y_{a},\alpha )=-\text{log }[{\left(\frac{(x-x_{c})\text{ cos }\alpha +(y-y_{c})\text{ sin }\alpha }{x_{a}}\right)}^{2}\\ +{\left(\frac{-(x-x_{c})\text{ sin }\alpha +(y-y_{c})\text{ cos }\alpha }{y_{a}}\right)}^{2}+\epsilon ]+\text{log}(1+\epsilon )\leq 0. \end{array}$$

The logarithm operation is applied to reduce the numerical range of the inequality, which helps with numerical stability of the subsequently described solver, and the constant ϵ is included to avoid singularities when (4) is evaluated for (*x, y*) → (*x*_*c*_, *y*_*c*_) (Bitar et al., 2019a).

Modeling static obstacles as ellipses poses a challenge for handling obstacles of various shapes, from e.g., electronic nautical charts (ENCs). Generic obstacle shapes can be approximated as a set of elliptical obstacles (Wu, 2019), although this may require a large number of constraints for complex environments. Alternatively, the obstacle modeling can be modified to allow for generic shapes. Zhang et al. (2018) present an interesting solution to handle polygon-shaped obstacles by introducing a signed distance function in the optimization problem. Unfortunately, this approach introduces a large number of slack variables and constraints, limiting feasibility for more than a few static obstacles.

### 3.2. Trajectory Generation and Optimization

From a scenario consisting of static obstacles, as mentioned in section 3.1, we find the piecewise linear shortest path by performing an A^⋆^ search on a uniformly decomposed grid. The resulting path is converted to a time-parameterized full-state trajectory by assuming a constant forward velocity, and connecting the shortest path with straight segments and circle arcs. The constant forward velocity is

(5) $$u_{\text{nom}}=\frac{L_{\text{path}}}{t_{\text{max}}},$$

where *L*_path_ is the length of the connected path, and *t*_max_ is the maximum allowed time to complete the trajectory. This full-state trajectory is then used as an initial guess to solve the OCP that gives the energy-optimized trajectory:

(6a) $$\begin{array}{l} \underset{\text{z}(\cdot ),\tau (\cdot )}{\text{min}}\int_{0}^{t_{\text{max}}}F_{\text{hi}}(\text{z}(t),\tau (t))\text{d}t \end{array}$$

subject to

(6b) $$\dot{\text{z}}(t)=\text{f}(\text{z}(t),\tau (t))\forall t\in [0,t_{\text{max}}]$$

(6c) $${\text{h}}_{\text{hi}}(\text{z}(t),\tau (t))\leq 0\forall t\in [0,t_{\max}]$$

(6d) $${\text{e}}_{\text{hi}}(\text{z}(0),\text{z}(t_{\text{max}}))=0.$$

The solution of this OCP is a trajectory of states ***z***(·) and inputs **τ**(·) that minimizes the cost functional in (6a). The ASV model from section 2 is rewritten as $\overset{∙}{\text{z}}=\text{f}\left(\text{z},\tau \right)$, where $\text{z}={\left[\eta^{⊤},{\text{x}}_{r}^{⊤}\right]}^{⊤}$ and ***f***(***z***, **τ**) represents (1).

The cost functional (6a) is chosen to minimize energy. The cost-to-go function is

(7) $$F_{\text{hi}}(\text{z},\tau )=K_{e}F_{e}(\text{z},\tau )+K_{\delta }\tau_{\delta }^{2},$$

with tuning parameters *K*_*e*_, *K*_δ_ > 0. The first term consists of a function that is proportional to mechanical work performed by the ASV:

(8) $$F_{e}(\text{z},\tau )=|\underset{\propto \text{ surge force}}{\underset{︸}{n{\left(\tau_{m}\right)}^{2}\cdot \text{cos }\delta (\tau_{\delta })}}\cdot u_{r}|+|\underset{\propto \text{ yaw moment}}{\underset{︸}{n{\left(\tau_{m}\right)}^{2}\cdot \text{sin }\delta (\tau_{\delta })\cdot L_{m}}}\cdot r|.$$

The function *n*: ℝ^+^ → ℝ^+^ maps the control input τ_*m*_ to propeller angular velocity. The function δ: ℝ → S maps the control input τ_δ_ to outboard motor angle. The second term in (8) is a quadratic cost to yaw control, included to avoid issues with singularity when solving the OCP.

The inequality constraints (6c) observe state boundaries as well as the static obstacles as represented in section 3.1. The boundary conditions (6d) denote initial and final constraints, i.e., start and end states.

A detailed description of the transcription of the OCP (6) to a non-linear program (NLP) using multiple shooting with *N*_hi_ shooting intervals is found in Bitar et al. (2019b).

## 4. Mid-Level COLAV

The mid-level algorithm, initially presented in Eriksen and Breivik (2017b) and further developed in Bitar et al. (2019a), is a model predictive control (MPC)-based algorithm intended for long-term COLAV. The algorithm utilizes gradient-based optimization, and takes both static and moving obstacles into account while attempting to follow an energy-optimized nominal trajectory from the high-level planner. The algorithm produces maneuvers complying with Rule 8 of the COLREGs, which requires maneuvers to be made in ample time and be readily observable for other vessels. The optimization problem is formulated as a NLP, which gives flexibility in designing the optimization problem.

In this section, the algorithm is extended to also consider COLREGs rules 13–16 and the first part of Rule 17.

### 4.1. The International Regulations for Preventing Collisions at Sea (COLREGs)

The COLREGs consists of a total of 37 rules and is divided into five parts (Cockcroft and Lameijer, 2004), where part B (rules 4–19) contains relevant rules on the conduct of vessels in proximity of each other. The most relevant rules for designing COLAV systems in part B are rules 8 and 13–17:

**Table:** 

**Rule 8**	**Action to avoid collision**. This rule states that actions taken to avoid collision should be large enough to be readily observable of other ships, implying that series of small alternations in speed and/or course should not be applied. The rule also recommends that course changes should be prioritized over speed changes if there is enough free space available, and that maneuvers must be made in ample time.
**Rule 13**	**Overtaking**. This rule states that a vessel is overtaking another if it approaches the other vessel with a course more than 22.5° abaft her beam. The overtaking vessel has to stay clear of the overtaken vessel, but there is no statements on which side of the vessel one should pass.
**Rule 14**	**Head on**. When two power-driven vessels approach each other on reciprocal, or nearly reciprocal, courses, they are in a head-on situation. In such a situation, both vessels should change their course to starboard, passing each other port-to-port, as shown in Figure 3A. This rule states no explicit definition on what should be considered to be reciprocal, or nearly reciprocal, courses, but court decisions indicate head-on situations exist for opposing courses ±6°. Notice that the rule does not include sailing vessels, which are covered by Rule 12.
**Rule 15**	**Crossing**. When two vessels approach each other such that the situation is not a head on or an overtaking, it is a crossing situation. The vessel with the other one to her starboard side is deemed the give-way vessel, while the other vessel is deemed the stand-on vessel. As shown in Figure 3B, the give-way vessel should maneuver to avoid collision, preferably by passing behind the stand-on vessel, while the stand-on vessel should keep her speed and course.
**Rule 16**	**Action by the give-way vessel**. Every vessel which is required to keep out of the way of another vessel should take early and large enough action to safely avoid collision.
**Rule 17**	**Action by the stand-on vessel**. This rule requires that a stand-on vessel should keep its current speed and course. The stand-on vessel may, however, maneuver to avoid collision if it becomes apparent that the give-way vessel is not taking appropriate actions to avoid collision. Furthermore, if the stand-on vessel finds itself so close to the obstacle that collision can not be avoided by the give-way vessel alone, the stand-on vessel should take such action which best aids to avoid collision. In a crossing situation, the stand-on vessel should avoid maneuvering to port, since this could lead to a collision if the give-way vessel maneuvers to starboard.

In the hybrid architecture illustrated in Figure 1, the mid-level algorithm is given the task of strictly enforcing COLREGs rules 13–16 and the stand-on requirement of Rule 17, while also complying with Rule 8.

In addition, we want the mid-level algorithm to comply with the first part of Rule 17, by not maneuvering to avoid collision in crossing situations if the ownship is the stand-on vessel. The hybrid COLAV system is inherently capable of adhering to the remaining requirement of Rule 17, where the stand-on vessel is allowed or required to maneuver, by having different prediction horizons and safety margins in the mid-level and short-term layers. The BC-MPC algorithm does not have any limitations of not maneuvering in stand-on situations, and will hence maneuver in stand-on situations if we come sufficiently close to the obstacle.

The mid-level algorithm as presented in Bitar et al. (2019a) only complies with Rule 8. Further in this section, we therefore present improvements to the mid-level algorithm to make it comply with rules 13–16 and the stand-on requirement of Rule 17.

### 4.2. COLREGs Interpretation

A commonly used concept for interpreting obstacles in COLAV algorithms is to assign a spatial region to obstacles, which the ownship should not enter. This approach is commonly referred to as a *domain-based* approach. Specially designed ship domains are commonly used for interpreting the COLREGs in COLAV algorithms, where the required clearance to an obstacle is significantly larger if the maneuver violates the COLREGs (Szlapczynski and Szlapczynska, 2017; Eriksen et al., 2019). This approach is attractive since it continuously captures multiple COLREGs rules, and does not require logic or discrete decisions. However, such an approach does not strictly enforce the COLREGs rules, since it will allow maneuvers violating the rules if they are large enough. In addition, a ship-domain approach will not be able to strictly enforce the stand-on requirement of Rule 17, since a domain-based approach will avoid collision with all obstacles. One could ignore obstacles with give-way obligations, but this would require an explicit COLREGs interpretation which conflicts with domain-based approaches' core idea of implicit COLREGs interpretation. Therefore, we pursue an alternative approach to handling the COLREGs in the mid-level algorithm.

To simplify the COLREGs interpretation task, we look at the situation from a static perspective, assuming that the current COLREGs situations are valid throughout the entire prediction horizon of the mid-level algorithm. In reality, the COLREGs situations may, however, change during the prediction horizon depending on both the ownship's and obstacles future trajectory. For instance, an obstacle approaching from head on, but far enough away to not be considered as a danger may be put in a safe state. Hence, the mid-level algorithm will (for the current iteration) act like no COLREGs rule applies to this vessel for the entire prediction horizon, while the obstacle may get close enough during the prediction horizon to be considered as a head-on situation. An MPC scheme of only implementing a small part of the prediction horizon will reduce the implications of this, since the situation is reassessed each time mid-level algorithm is run, which justifies the assumption of considering the COLREGs from a static perspective. Investigating the possibilities for dynamically predicted future COLREGs situations as part of the MPC prediction will be considered as future work.

#### 4.2.1. State Machine

We propose to utilize a state machine in order to decide which COLREGs rule is active with respect to each obstacle in the vicinity of the ownship. The state machine contains the states:

**Table:** 

**SF**	Safe state. This implies that the COLREGs do not enforce any rule with respect to this obstacle.
**OT**	Overtaking state. This implies that COLREGs Rule 13 applies with respect to this obstacle. The state machine does not discriminate on whether the ownship is overtaking another vessel or is being overtaken, but this can be done by looking at which vessel has the higher speed (Tam and Bucknall, 2010).
**HO**	Head-on state. This implies that COLREGs Rule 14 applies with respect to this obstacle.
**GW**	Give-way state. This implies that COLREGs Rule 15 applies with respect to this obstacle, and the ownship has to give way.
**SO**	Stand-on state. This implies that COLREGs Rule 15 applies with respect to this obstacle, and the ownship has to stand on.
**EM**	Emergency state. This implies that the obstacle is so close and/or behaves unpredictably, such that special considerations must be made.

As shown in Figure 4, all transitions have to go either from or to the safe state.

**Figure 3:**
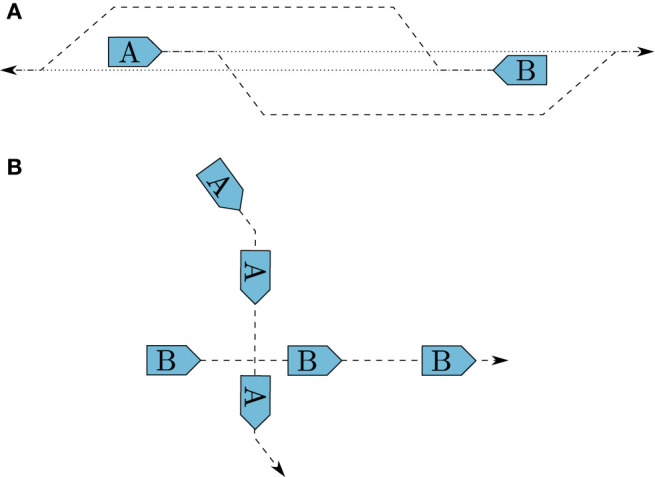
Illustration of head-on **(A)** and crossing **(B)** situations, and how they should be resolved.

**Figure 4:**
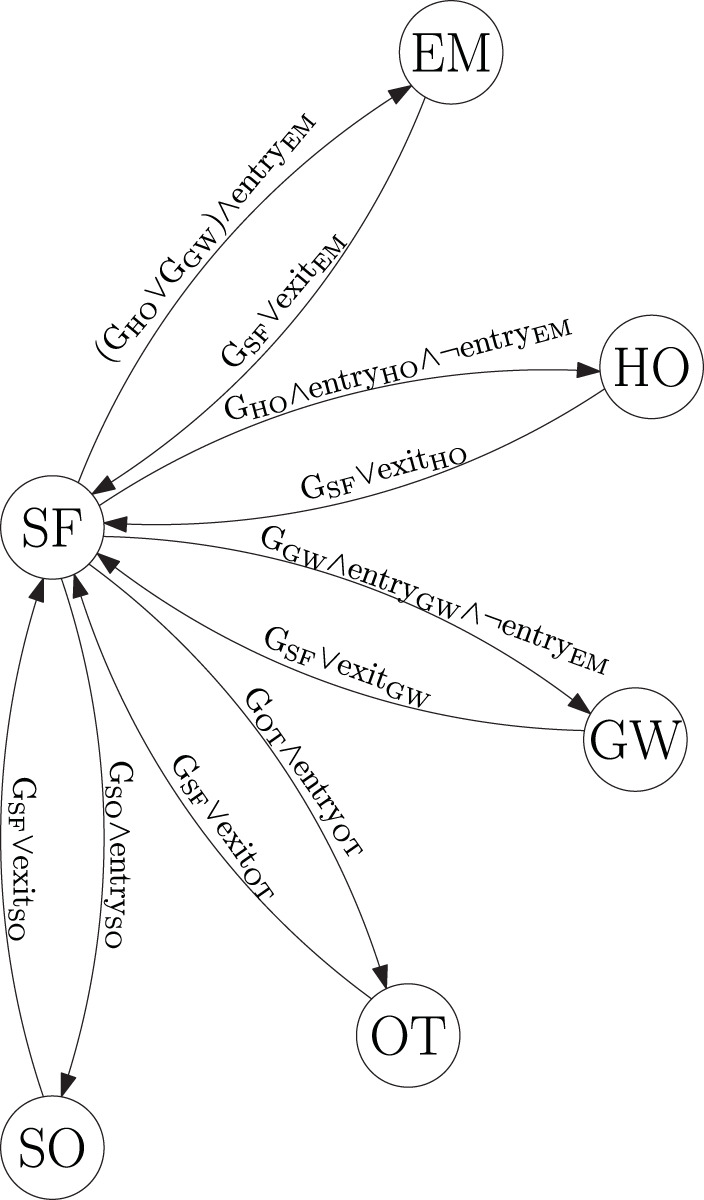
COLREGs state machine. The abbreviations “G_SF_,” “G_SO_,” “G_OT_,” “G_GW_,” and “G_HO_” denote geometrical situations, while “entry_xx_” and “exit_xx_” denote additional state-dependent entry and exit criterias.

This implies that when the state machine decides that a COLREGs (or emergency) situation exists with respect to an obstacle, it will not allow switching to another state without the situation being considered as safe first. One could argue that it should be able to transition between specific states, like e.g., from head-on, give-way and overtaking to emergency. This is an interesting topic, which should receive attention in the future. To control the transitions between the different states, we combine the time to and distance at the CPA, a CPA-like measure of the time until a critical point and a geometrical interpretation of the situation.

#### 4.2.2. Entry and Exit Criteria

CPA is a common concept in maritime risk assessment. Given the current speed and course of the ownship and an obstacle, CPA describes the time to the point where the two vessels are the closest, and the distance to the obstacle at this point. Given the position and velocity vector of the ownship ***p***, ***v*** and an obstacle ***p***_*o*_, ***v***_*o*_, the time to CPA is calculated as (Kufoalor et al., 2018)

(9) $$t_{\text{CPA}}=\{\begin{array}{ll} 0 & \text{if }{\left\|\text{v}-{\text{v}}_{0}\right\|}_{2}\leq \epsilon \\ \frac{\left(\text{p}-{\text{p}}_{o})\cdot (\text{v}-{\text{v}}_{o}\right)}{{\left\|\text{v}-{\text{v}}_{o}\right\|}_{2}^{2}} & \text{else}, \end{array}$$

where ϵ > 0 is a small constant in order to avoid division by zero in the case where the relative velocity between the ownship and obstacle is zero. Given *t*_CPA_, we calculate the distance between the vessels at CPA as

(10) $$d_{\text{CPA}}={\left\|\left(\text{p}+t_{\text{CPA}}\text{v})-({\text{p}}_{o}+t_{\text{CPA}}{\text{v}}_{o}\right)\right\|}_{2}.$$

While the CPA is the point where the distance to an obstacle is at its minimum, the critical point is where the distance to an obstacle crosses underneath a critical distance *d*_crit_. This critical distance describes a minimum obstacle distance that the mid-level algorithm is designed for. The time to the critical point *t*_crit_ can be calculated by solving the equation

(11) $${\left\|\left(\text{p}+t_{\text{crit}}\text{v})-({\text{p}}_{o}+t_{\text{crit}}{\text{v}}_{o}\right)\right\|}_{2}=d_{\text{crit}}.$$

In the cases where the distance between the ships does not fall below *d*_crit_, *t*_crit_ is undefined. Otherwise, there are generally two solutions. The interesting solution is the one with the lowest *t*_crit_ value, as this is when the obstacle enters the *d*_crit_ boundary.

The state-machine entry criteria in Figure 4 are defined as

(12) $$\begin{array}{l} {\text{entry}}_{i}=\{\begin{array}{ll} \text{true} & \text{if }d_{\text{CPA}}<{\bar{d}}_{\text{CPA}}^{i,\text{enter}}\wedge t_{\text{CPA}}\in [{\underline{t}}_{\text{ CPA}}^{i,\text{enter}},{\bar{t}}_{\text{CPA}}^{i,\text{enter}}],\\ \text{false} & \text{otherwise} \end{array}\\ \;\forall i\in \{\text{SO},\text{OT},\text{GW},\text{HO}\}\\ {\text{entry}}_{\text{EM}}=\{\begin{array}{ll} \text{true} & \text{if }t_{\text{crit}}<{\bar{t}}_{\text{crit}}^{\text{EM},\text{enter}}\wedge t_{\text{CPA}}>0\\ \text{false} & \text{otherwise}, \end{array} \end{array}$$

where ${\bar{d}}_{\text{CPA}}^{i,\text{enter}},{\underline{t}}_{\text{CPA}}^{i,\text{enter}}$, and ${\bar{t}}_{\text{CPA}}^{i,\text{enter}}$ for *i* ∈ {SO, OT, GW, HO} are tuning parameters denoting thresholds on *d*_CPA_ and *t*_CPA_ in order to satisfy the entry criteria for the stand-on, overtaking, give-way and head-on states. The tuning parameter ${\bar{t}}_{\text{crit}}^{\text{EM,enter}}$ denotes an upper limit on *t*_crit_ in order to enter the emergency state. The idea behind the stand-on, overtaking, give-way and head-on entry criterias are that in order for the obstacle to represent a risk, both *t*_CPA_ and *d*_CPA_ need to be within some tunable thresholds. Situations with a very low *d*_CPA_, but with a high *t*_CPA_, will not trigger the entry criteria, since the situations will not occur in the near future. Similarly, if *t*_CPA_ is within the thresholds, but *d*_CPA_ is large, this indicates a safe passing where risk of collision does not exist. The lower bound on *t*_CPA_ will typically be selected as zero, and is useful to distinguish between obstacles moving toward of away from the ownship. For the emergency state, the entry criteria is based on the critical point, at which we are so close that the mid-level algorithm may struggle with providing meaningful maneuvers. In addition to *t*_crit_ being under the threshold ${\bar{t}}_{\text{crit}}^{\text{EM,enter}}$, we require that *t*_CPA_ is positive, indicating that we are getting closer to the obstacle. Currently, we only allow entering the emergency state if the situation is a geometrical give-way or head-on, since an overtaking situation represents a smaller danger and has less requirement for special consideration.

The state-machine exit criterias in Figure 4 are defined as

(13) $$\begin{array}{l} \;{\text{exit}}_{i}=\{\begin{array}{ll} \text{true} & \text{if }d_{\text{CPA}}\geq {\underline{d}}_{\text{CPA}}^{i,\text{exit}}\vee t_{\text{CPA}}\notin [{\underline{t}}_{\text{CPA}}^{i,\text{exit}},{\bar{t}}_{\text{CPA}}^{i,\text{exit}}],\\ \text{false} & \text{otherwise} \end{array}\\ \;\forall i\in \{\text{SO},\text{OT},\text{GW},\text{HO}\}\\ {\text{exit}}_{\text{EM}}=\{\begin{array}{ll} \text{true} & \text{if }t_{\text{crit}}\geq {\underline{t}}_{\text{ crit}}^{\text{EM},\text{exit}}\vee t_{\text{CPA}}\leq 0\\ \text{false} & \text{otherwise}, \end{array} \end{array}$$

where ${\underline{d}}_{\text{CPA}}^{i,\text{exit}},{\underline{t}}_{\text{CPA}}^{i,\text{exit}}$, and ${\bar{t}}_{\text{CPA}}^{i,\text{exit}}$ for *i* ∈ {SO, OT, GW, HO} are tuning parameters denoting thresholds on *d*_CPA_ and *t*_CPA_ in order to satisfy the exit criteria for the stand-on, overtaking, give-way and head-on states. The exit criteria for the emergency state is satisfied if *t*_crit_ is larger than the tuning parameter ${\bar{t}}_{\text{exit}}^{\text{EM,enter}}$, or *t*_CPA_ is negative, implying that the obstacle is moving further away from the ownship. Note that the exit criterias are obtained by negating the entry criterias, but with other thresholds in order to implement hysteresis to avoid shattering. In general, we allow for different tuning parameters for the different states, but in our simulations we see that selecting the same tuning parameters for all states provides good results. Therefore, we define:

(14) $$\begin{array}{l} {\bar{d}}_{\text{CPA}}^{i,\text{enter}}={\bar{d}}_{\text{CPA}}^{\text{enter}}\\ {\underline{t}}_{\text{CPA}}^{i,\text{enter}}={\underline{t}}_{\text{CPA}}^{\text{enter}}\\ {\bar{t}}_{\text{CPA}}^{i,\text{enter}}={\bar{t}}_{\text{CPA}}^{\text{enter}}, \end{array}$$

and

(15) $$\begin{array}{l} {\underline{d}}_{\text{CPA}}^{i,\text{exit}}={\underline{d}}_{\text{CPA}}^{\text{exit}}\\ {\underline{t}}_{\text{CPA}}^{i,\text{exit}}={\underline{t}}_{\text{CPA}}^{\text{exit}}\\ {\bar{t}}_{\text{CPA}}^{i,\text{exit}}={\bar{t}}_{\text{CPA}}^{\text{exit}} \end{array}$$

for all *i* ∈ {SO, OT, GW, HO}.

#### 4.2.3. Geometrical Situation Interpretation

**Figure 5:**
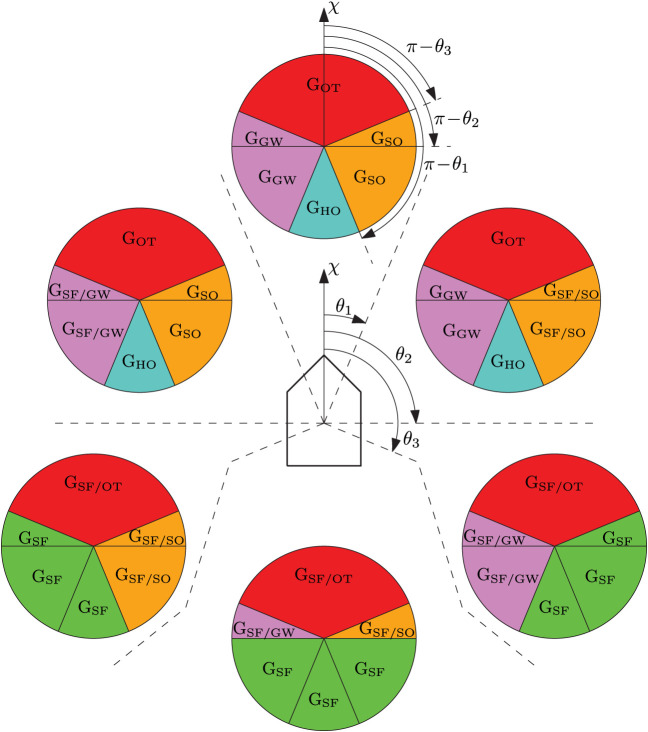
Illustration of the geometrical COLREGs interpretation, where the ownship course is denoted as χ and θ_1_, θ_2_, θ_3_ denote symmetrical regions given as [22.5, 90, 112.5°] offset from ahead. The circles illustrate obstacles in different relative bearing regions, and have a fixed orientation with respect to the ownship. The geometrical situations are color-coded and denoted as G_*i*_, *i* ∈ {SF, SO, OT, GW, HO} for safe, stand-on, overtaking, give-way and head-on situations, respectively. When two situations are given, like e.g., G_SF/SO_, we use the former (SF) if *t*_CPA_ < 0 and the latter (SO) if *t*_CPA_ ≥ 0, analogous to the obstacle moving away or toward the ownship. To decide the geometrical situation, we first find which relative bearing region the obstacle resides in, before finding which obstacle region the obstacle's course resides in. The figure is inspired by Tam and Bucknall (2010).

Tam and Bucknall (2010) present a geometrical interpretation scheme for deciding COLREGs situations based on the relative position, bearing and course of the obstacle with respect to the ownship. We base our geometrical interpretation on a slightly modified version of this scheme, where we include the sign of *t*_CPA_ to distinguish between situations where the obstacle moves closer toward or farther away from the ownship. The geometrical interpretation is shown in Figure 5, where the geometrical situation is obtained by finding which region the obstacle position and course resides in.

Notice that the head-on region is larger than the threshold of ±6° as described by the COLREGs. The reason for this is that Tam and Bucknall recommend using a larger region of 22.5° in order to increase the robustness of the geometrical COLREGs interpretation scheme.

### 4.3. Interface to the High-Level Planner

The high-level planner produces an energy-optimized nominal trajectory for the ownship to follow. However, since the high-level planner does not consider moving obstacles, the speed is the only time-relevant factor of the desired trajectory. In a case where the ownship for some reason, e.g., avoiding moving obstacles, lag behind the nominal trajectory, following the nominal trajectory in absolute time would cause a speed increase in order to catch up with it. Therefore, the mid-level algorithm performs *relative trajectory tracking*, where it tracks the nominal trajectory with a time offset *t*_*b*_ ∈ ℝ. This results in a relative nominal trajectory for the mid-level algorithm:

(16) $${\bar{\text{p}}}_{d}(t)={\text{p}}_{d}(t+t_{b}),$$

where ${\text{p}}_{d}={\left[N_{d}\left(t\right),E_{d}\left(t\right)\right]}^{⊤}$ is the nominal trajectory from the high-level planner. The time offset *t*_*b*_ is calculated each time the mid-level algorithm is run by solving a separate optimization problem, and is selected such that ${\bar{\text{p}}}_{d}\left(t_{0}\right)$ is the point on the nominal trajectory closest to the ownship. See Bitar et al. (2019a) for a detailed description of this concept.

### 4.4. Optimization Problem Formulation

The mid-level algorithm is formalized as an OCP:

(17a) $$\underset{\eta (\cdot ),{\text{x}}_{r}(\cdot )}{\text{min}}\phi (\eta (\cdot ),{\text{x}}_{r}(\cdot ))$$

subject to

(17b) $$\dot{\eta }(t)=\text{R}(\psi (t)){\text{x}}_{r}(t)+\left[\begin{array}{c} {\text{V}}_{c}\\ 0 \end{array}\right]\forall t\in [t_{0},t_{0}+T_{h}]$$

(17c) $${\text{h}}_{\text{mid}}(\eta (t),{\text{x}}_{r}(t),t)\leq 0\forall t\in [t_{0},t_{0}+T_{h}]$$

(17d) $${\text{e}}_{\text{mid}}(\eta (t_{0}))=0,$$

where *T*_*h*_ > 0 is the prediction horizon, ϕ(·, ·) is the objective functional, (17b) contains a kinematic vessel model, (17c) contains inequality constraints and (17d) contains boundary constraints.

Analytical solutions of OCPs are in general not possible to find. A more common approach is to transcribe the OCP to an NLP, and solve that using a gradient optimization scheme. In our case, we transcribe (17) into an NLP with *N*_*p*_ samples using multiple shooting, where the vessel model is discretized using 4th order Runge Kutta and the cost functional is discretized using forward Euler. The resulting NLP is given as

(18) $$\begin{array}{l} \underset{\text{w},\omega ,\mu ,\xi }{\text{min}}\phi_{p}(\text{w},\omega ,\mu )+\phi_{c}(\text{w})+\phi_{\text{COLREGs}}(\text{w})+\phi_{\xi }(\xi )\\ \text{subject to}\\ \text{g}(\text{w},\eta (t_{0}))=0\\ \text{h}(\text{w},\xi )\leq 0\\ {\bar{\text{h}}}_{k}(\eta_{k},\omega_{k},\mu_{k},{\bar{\text{p}}}_{d,k})\leq 0\forall k\in \{1,\ldots ,N_{p}\}\\ \xi \geq 0, \end{array}$$

where $\text{w}={\left[\eta_{0}^{⊤},{\text{x}}_{r,0}^{⊤},\ldots ,\eta_{N_{p}-1}^{⊤},{\text{x}}_{r,N_{p}-1}^{⊤},\eta_{N_{p}}^{⊤}\right]}^{⊤}\in {\mathbb{R}}^{5N_{p}+3}$ is a vector of 5*N*_*p*_+3 decision variables and ${\bar{\text{p}}}_{d,1:N_{p}}=\left[{\bar{\text{p}}}_{d,1},{\bar{\text{p}}}_{d,2},\ldots ,{\bar{\text{p}}}_{d,N_{p}}\right]$ is a sequence of desired positions. The vectors $\omega \in {\mathbb{R}}^{2N_{p}}$, $\mu \in {\mathbb{R}}^{2N_{p}}$ and $\xi \in {\mathbb{R}}^{MN_{p}}$ contain slack variables, where *M* is the number of moving obstacles to be included in the constraints.

The vector $\text{g}\left(\text{w},\eta \left(t_{0}\right)\right)\in {\mathbb{R}}^{3N_{p}+3}$ contains shooting and boundary constraints, while $\text{h}\left(\text{w}\right)\in {\mathbb{R}}^{\left(M+D+4\right)N_{p}}$, where *D* is the number of static obstacles, contain inequality constraints ensuring COLAV and steady-state vessel velocity feasibility. The vectors ${\bar{\text{h}}}_{k}\left(\eta_{k},\omega_{k},\mu_{k},{\bar{\text{p}}}_{d,k}\right)\in {\mathbb{R}}^{6}$, *k* ∈ {1, *N*_*p*_} contain constraints on the slack variables **ω** and **μ**.

In the following subsections, we describe the terms in (18) in more detail.

#### 4.4.1. Objective Function

The objective function contains four functions, where ϕ_*p*_(***w***, **ω**, **μ**) introduces cost on deviating from the relative nominal trajectory ${\bar{\text{p}}}_{d}\left(t\right)$, ϕ_*c*_(***w***) introduces cost on using control input, ϕ_COLREGs_(***w***) is a COLREGs-specific function and ϕ_ξ_(**ξ**) introduces slack variable cost.

To avoid that the NLP changes behavior when moving away from the nominal trajectory, we wish to have linear growth in the position error function ϕ_*p*_(***w***, **ω**, **μ**). This is achieved by instead of using quadratic terms in the position error function, we use the Huber loss function which is quadratic around the origin and resembles the absolute value function above a given threshold σ > 0:

(19) $$H(\rho )=\{\begin{array}{ll} \frac{1}{2}\rho^{2} & \;\left|\rho \right|\leq \sigma \\ \sigma (\left|\rho \right|-\frac{1}{2}\sigma ) & \;\left|\rho \right|>\sigma . \end{array}$$

The Huber loss function has a discontinuous gradient, making it slightly complicated to implement in gradient-based optimization problems. It can, however, be implemented in a continuous fashion by utilizing lifting, where slack variables are introduced to create a problem of a higher dimensionality which is easier to solve. Using this technique, ${\bar{\phi }}_{p}\left(\text{w},\omega ,\mu \right)$ is defined as

(20) $${\bar{\phi }}_{p}(\text{w},\omega ,\mu )=K_{p}\sum_{k\;=\;1}^{N_{p}}\sigma \;1{\;}^{⊤}\omega_{k}+\frac{1}{2}\mu_{k}^{⊤}\mu_{k},$$

where *K*_*p*_ > 0 is a tuning parameter, and $\omega_{k}\in {\mathbb{R}}^{2}$ and $\mu_{k}\in {\mathbb{R}}^{2}$ are slack variables constrained by

(21) $$\begin{array}{l} {\bar{\text{h}}}_{k}(\text{w},\omega ,\mu ,{\bar{\text{p}}}_{d,k})=\left[\begin{array}{c} {\text{v}}_{\text{k}}+\mu_{\text{k}}+{\text{p}}_{k}-{\bar{\text{p}}}_{d,k}\\ {\text{v}}_{\text{k}}+\mu_{\text{k}}-({\text{p}}_{k}-{\bar{\text{p}}}_{d,k})\\ \;-\omega_{k} \end{array}\right]\leq 0\\ \;\forall k\in \{1,\ldots ,N_{p}\}, \end{array}$$

where ***p***_*k*_ is the predicted vessel position at time step *k*, i.e., $\eta_{k}={\left[{\text{p}}_{k}^{⊤},\psi_{k}\right]}^{⊤}$. See Bitar et al. (2019a) for more details.

Rule 8 of the COLREGs requires that maneuvers are readily observable for other vessels, implying that speed and course changes should have a sufficiently large magnitude, and not be performed as a sequence of small changes. In order to enforce this in the optimization problem, the control cost function ϕ_*c*_(***w***) introduces a non-linear cost on the change in speed and course, which makes the algorithm favor readily observable maneuvers. The function is defined as

(22) $$\phi_{c}(\text{w})=\sum_{k\;=\;0}^{N_{p}-1}K_{\dot{U}}q_{\dot{U}}({\dot{U}}_{k})+K_{\dot{\chi }}q_{\dot{\chi }}({\dot{\chi }}_{k}),$$

where $K_{\overset{∙}{U}},K_{\overset{∙}{\chi }}>0$ are tuning parameters, while $q_{\overset{∙}{U}}\left({\overset{∙}{U}}_{k}\right)$ and $q_{\overset{∙}{\chi }}\left({\overset{∙}{\chi }}_{k}\right)$ are the non-linear cost functions. Notice that neither the speed over ground (SOG) *U* nor the course χ are elements of the search space, but they can be computed as $U=\sqrt{u^{2}+v^{2}}$ and $\chi =\psi +\arcsin\frac{v}{U}$. Their derivatives are then calculated by finite differencing. See Eriksen and Breivik (2017a) and Bitar et al. (2019a) for more details on the control cost function.

The ϕ_COLREGs_(***w***) function introduces a COLREGs-specific cost with respect to obstacles based on the rule currently applicable as defined by the state machine. We hence tailor the NLP to the current situation. The function is defined as

(23) $$\begin{array}{l} \phi_{\text{COLREGs}}(\text{w})=\sum_{k=1}^{N_{p}}[\sum_{i\in O_{\text{HO}}}K_{\text{HO}}V_{\text{HO},i,k}({\text{p}}_{k})+\sum_{i\in O_{\text{GW}}}K_{\text{GW}}V_{\text{GW},i,k}({\text{p}}_{k})\\ \;\;+\sum_{i\in O_{\text{SO}}}K_{\text{SO}}V_{\text{SO},k}(\text{w})+\sum_{i\in O_{\text{EM}}}K_{\text{EM}}V_{\text{EM},k}(\text{w})]\;, \end{array}$$

where $O_{\text{HO}},O_{\text{GW}},O_{\text{SO}}$, and $O_{\text{EM}}$ contain obstacles which are in the head-on, give-way, stand-on and emergency states, respectively, and *K*_HO_, *K*_GW_, *K*_SO_, *K*_EM_ > 0 are tuning parameters. The functions *V*_HO, *i, k*_(***p***_*k*_), *V*_GW, *i, k*_(***p***_*k*_), *V*_SO, *k*_(***w***), and *V*_EM, *k*_(***w***) describe functions capturing head-on, give-way, stand-on and emergency behavior with respect to obstacle *i*, respectively. Notice that the head-on and give-way functions vary with both the obstacle number and time step number, which is due to the functions depending on the given obstacles position and course at time step *k*.

**Figure 6:**
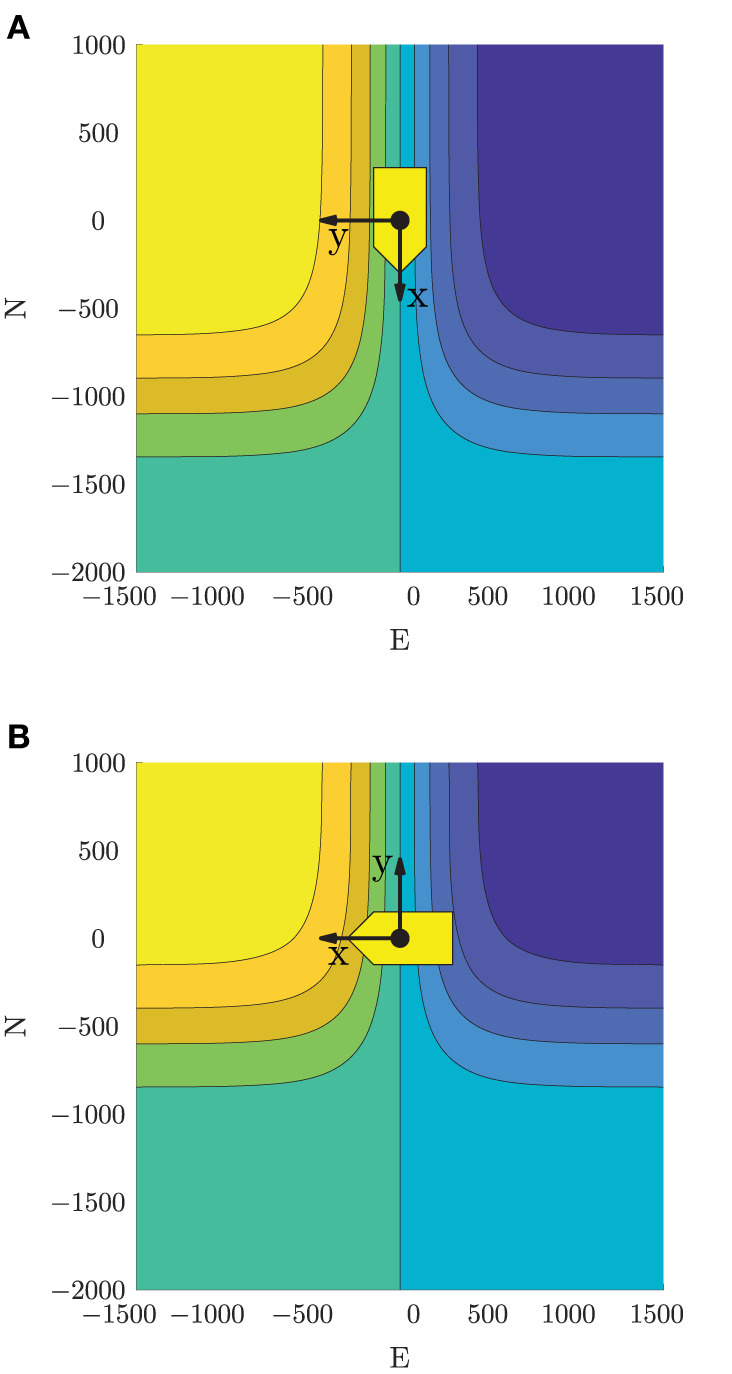
Potential functions ensuring passing on the correct side in head-on and give-way situations. Yellow indicates a positive value, blue indicates a negative value, while the yellow patch and axis cross show the obstacle location and course-fixed coordinate system. Used in a minimization scheme, this will favor starboard maneuvers in head-on situations, and passing behind obstacles in give-way situations. Note that the obstacle here has zero sideslip, resulting in the heading and course pointing in the same direction. **(A)** Head-on potential function. **(B)** Give-way potential function.

For head-on situations, we define a potential function with a positive value on the obstacle's starboard side, and a negative value on its port side. When used in the objective function, this will favor trajectories passing a head-on obstacle on its port side, in compliance with Rule 14 of the COLREGs. In addition, the potential function has an attenuation term, reducing the impact of the function when far away from an obstacle:

(24) $$V_{\text{HO},i,k}(\text{p})=\frac{\text{tanh}\left(\alpha_{x,\text{HO}}(x_{0,\text{HO}}-x^{\left\{i,k\right\}})\right)}{2}\text{tanh}(\alpha_{y,\text{HO}}y^{\left\{i,k\right\}})\in (-1,1),$$

where α_*x*, HO_, α_*y*, HO_ > 0 are tuning parameters controlling the steepness of the head-on potential function and ${\bar{x}}_{0,\text{HO}}>0$ is a tuning parameter controlling the influence of the attenuating potential. The coordinate (*x*^{*i, k*}^, *y*^{*i, k*}^) is ***p*** given in obstacles *i*'s course-fixed frame (in which the *x*-axis points along the obstacle's course) at time step *k*, computed as

(25) $$\left[\begin{array}{c} x^{\left\{i,k\right\}}\\ y^{\left\{i,k\right\}} \end{array}\right]=\text{R}{\left(\chi_{i,k}\right)}^{⊤}\left(\text{p}-{\text{p}}_{o,k,i}\right),$$

where ***p***_*o, k, i*_ and χ_*i, k*_ are the position and course of obstacle *i* at time step *k*. The head-on potential function with parameters α_*x*, HO_ = 1/500, α_*y*, HO_ = 1/400 and *x*_0, HO_ = 1, 000 m is shown in Figure 6A.

For give-way situations, we define a similar potential function, but rotated such that the function is positive in front of an obstacle and negative behind it. This will favor trajectories passing behind an obstacle, as desirable with respect to Rule 15 when a give-way obligation is active. The give-way potential function is defined as

(26) $$V_{\text{GW},i,k}(\text{p})=\frac{\text{tanh}\left(\alpha_{y,\text{GW}}(y^{\left\{i,k\right\}}-y_{0,\text{GW}})\right)}{2}\text{tanh}(\alpha_{x,\text{GW}}x^{\left\{i,k\right\}})\in (-1,1),$$

where α_*x*, GW_, α_*y*, GW_ > 0 control the steepness of the give-way potential function and ȳ_0, GW_ < 0 control the attenuation on the port side of an obstacle. The give-way potential function with parameters α_*x*, GW_ = 1/400, α_*y*, GW_ = 1/500 and *y*_0, GW_ = −500 m is shown in Figure 6B.

In stand-on situations, we want the mid-level algorithm to disregard the obstacle and keep the current speed and course in order to comply with the first part of Rule 17. One could simply constrain the algorithm to not maneuver, but this would be perilous in situations where the ownship simultaneously finds itself in a head-on or give-way situation. In such a situation it would be of extra importance to choose readily observable maneuvers, and we therefore design the stand-on cost with the same terms as used in the control cost (22) to amplify the effect:

(27) $$V_{\text{SO},k}(\text{w})=K_{\dot{U}}q_{\dot{U}}({\dot{U}}_{k})+K_{\dot{\chi }}q_{\dot{\chi }}({\dot{\chi }}_{k}).$$

If an obstacle is in an emergency state, the obstacle is disregarded in the mid-level algorithm and left for the short-term algorithm to handle. In such a situation, it is important that the mid-level algorithm behaves predictable, and we therefore use the same cost function as for stand-on situations:

(28) $$V_{\text{EM},k}(\text{w})=V_{\text{SO},k}(\text{w}).$$

The slack variable **ξ** is used in a homotopy scheme, which we introduce to avoid getting trapped in local minima around moving obstacles. The homotopy scheme is described in further detail in section 4.5. The homotopy cost function ϕ_ξ_(**ξ**) introduces slack cost on **ξ**:

(29) $$\phi_{\xi }(\xi )=K_{\xi }1^{⊤}\xi ,$$

where *K*_ξ_ > 0 is iteratively increased as part of the homotopy scheme.

### 4.5. Obstacle Handling and Steady-State Feasibility

The inequality constraint ***h***(***w***, **ξ**) ≤ **0** ensures COLAV and steady-state feasibility with respect to actuator limitations.

Static obstacles are handled similarly as in the high-level algorithm, with (4) representing an elliptical obstacle with center (*x*_*c*_, *y*_*c*_), angle α and major and minor axes *x*_*a*_ and *y*_*a*_, respectively. The constraint (4) needs to be enforced at each time step. Hence, for the *i*-th static obstacle, we define the constraint

(30) $${\text{h}}_{s_{i}}(\text{w})=\left[\begin{array}{c} h_{o}(x_{1},y_{1},x_{c,i},y_{c,i},x_{a,i},y_{a,i},\alpha_{i})\\ h_{o}(x_{2},y_{2},x_{c,i},y_{c,i},x_{a,i},y_{a,i},\alpha_{i})\\ \vdots \\ h_{o}(x_{N_{p}},y_{N_{p}},x_{c,i},y_{c,i},x_{a,i},y_{a,i},\alpha_{i}) \end{array}\right]\leq 0.$$

Moving obstacles are handled in a similar fashion, but letting the ellipsis center position and angle be time varying. Obstacles in stand-on situations should, however, not be included in the constraints, since the mid-level algorithm is supposed to stand on in such situations. Moreover, if an obstacle has entered an emergency state, the obstacle is so close and behaving unpredictably that the mid-level algorithm should disregard it and leave it for the short-term layer. Hence, for the *i*-th moving obstacle not in a stand-on or an emergency situation, we define the constraint

(31) $${\text{h}}_{m_{i}}(\text{w})=\left[\begin{array}{c} h_{o}(x_{1},y_{1},x_{c,i,1},y_{c,i,1},x_{a,i},y_{a,i},\alpha_{i,1})\\ \vdots \\ h_{o}(x_{N_{p}},y_{N_{p}},x_{c,i,N_{p}},y_{c,i,N_{p}},x_{a,i},y_{a,i},\alpha_{i,N_{p}}) \end{array}\right]\leq 0,$$

where *x*_*c, i, k*_, *y*_*c, i, k*_, and α_*i, k*_ denote the position and course of the *i*-th moving obstacle at time step *k*.

Given *D* static obstacles and *M* obstacles not in stand-on or emergency situations, we define the constraint

(32) $${\text{h}}_{o}(\text{w},\text{ξ})=\left[\begin{array}{c} {\text{h}}_{s_{1}}(\text{w})\\ \vdots \\ {\text{h}}_{s_{D}}(\text{w})\\ {\text{h}}_{m_{1}}(\text{w})\\ \vdots \\ {\text{h}}_{m_{M}}(\text{w}) \end{array}\right]+\left[\begin{array}{c} 0\\ \text{ξ} \end{array}\right],$$

where we include slack variables **ξ** ≥ **0** on the moving obstacle constraints as part of a homotopy scheme. The reason for using homotopy is that NLP solvers in general only finds local minima, and can have issues with moving an initial guess “through” obstacles. Normally, this is not an issue, but for the mid-level algorithm the optimal solution can change drastically from one iteration to another. This can for instance happen if an obstacle enters a head-on or give-way state, where the solution can be trapped on the wrong side of an obstacle. In general, homotopy describes introducing an extra parameter which is iteratively adjusted in order to iteratively move a local solution toward a global solution (Deuflhard, 2011). In our homotopy scheme, we introduce slack variables on the moving obstacle constraints, which will allow solutions to travel through obstacles at the cost of a homotopy cost (29) scaled by the homotopy parameter *K*_ξ_. Initially, this is selected as a low value to have a high amount of slack on the moving obstacles, while it is iteratively increased toward *K*_ξ_ → ∞, which results in **ξ** = **0** and hence no slack on moving obstacles. Currently, we only introduce slack on moving obstacles, but slack should also be introduced to static obstacles if they are small enough for the algorithm to be able to pass on both sides, like e.g., rocks, navigational marks, etc.

Similarly as in Eriksen and Breivik (2017b) and Bitar et al. (2019a), we ensure steady-state feasible trajectories at each time step through a constraint ${\text{h}}_{{\text{x}}_{r,k}}\left({\text{x}}_{r,k}\right)\leq 0\in {\mathbb{R}}^{4}$, which captures the state constraint ${\text{x}}_{r}\in X_{r}$ at time step *k*. To ensure stead-state feasibility for the entire prediction horizon, we define the constraint

(33) $${\text{h}}_{{\text{x}}_{r}}(\text{w})=\left[\begin{array}{c} {\text{h}}_{{\text{x}}_{r,k}}({\text{x}}_{r,0})\\ {\text{h}}_{{\text{x}}_{r,k}}({\text{x}}_{r,1})\\ \vdots \\ {\text{h}}_{{\text{x}}_{r,k}}({\text{x}}_{r,N_{p}-1}) \end{array}\right]\leq 0.$$

Finally, the inequality constraints are combined as.

(34) $$\text{h}(\text{w},\xi )=\left[\begin{array}{c} {\text{h}}_{o}(\text{w},\xi )\\ {\text{h}}_{{\text{x}}_{r}}(\text{w}) \end{array}\right]\in {\mathbb{R}}^{(M+D+4)N_{p}}.$$

## 5. Short-Term COLAV

For the short-term layer, the branching-course model predictive control (BC-MPC) algorithm is used, which is a sample-based MPC algorithm intended for short-term ASV COLAV. The BC-MPC algorithm was initially developed in Eriksen et al. (2019), extended to also consider static obstacles in Eriksen and Breivik (2019) and is experimentally validated in several full-scale experiments using a radar-based system for detecting and tracking obstacles. The algorithm complies with COLREGs rules 8, 13, and the second part of Rule 17, while favoring maneuvers complying with the maneuvering aspects of rules 14 and 15. Notice that Rule 17 allows a ship to ignore the maneuvering aspects of rules 14 and 15 in situations where the give-way vessel does not maneuver. The obstacle clearance will be larger if the algorithm ignores the maneuvering aspects of rules 14 and 15, like e.g., passing in front of an obstacle in a crossing situation where the ownship is the give-way vessel. Moving obstacles are in general handled by the mid-level algorithm, making this applicable only in emergency situations and for obstacles detected so late that the mid-level algorithm is unable to avoid them.

The algorithm constructs a search space consisting of a finite number of trajectories, which each contain a sequence of maneuvers. The maneuvers are constructed using a dynamic model of the ownship and a set of acceleration motion primitives, resulting in feasible trajectories being specified to the vessel controller. For each maneuver, a discrete set of SOG and course accelerations are created as

(35) $$\begin{array}{l} {\dot{\text{U}}}_{\text{samples}}=\left\{{\dot{U}}_{1},{\dot{U}}_{2},\ldots ,{\dot{U}}_{N_{U}}\right\}\\ \ddot{\chi }{\;}_{\text{samples}}=\left\{{\ddot{\chi }}_{1},{\ddot{\chi }}_{2},\ldots ,{\ddot{\chi }}_{N_{\chi }}\right\}, \end{array}$$

where ${\overset{∙}{U}}_{i},i\in \left[1,N_{U}\right]$ and ${\ddot{\chi }}_{i},i\in \left[1,N_{\chi }\right]$ denote *N*_*U*_ ∈ ℕ and *N*_χ_ ∈ ℕ vessel-feasible speed and course accelerations. Given the acceleration samples (35) and motion primitives for each maneuver in a trajectory, we create a set of desired SOG and course trajectories $U_{d}$. These trajectories have continuous acceleration, and is designed in an open-loop fashion by using the current reference tracked by the vessel controller for initialization, rather than the current vessel SOG and course. The reason for this is that the reference to the vessel controller should be continuous in order to avoid jumps in the actuator commands. To include feedback in the trajectory prediction, a set of feedback-corrected SOG and course trajectories ${\bar{U}}_{d}$ is predicted using a simplified error model of the vessel and vessel controller. Finally, the feedback-corrected SOG and course trajectories are used to compute a set of feedback-corrected pose trajectories:

(36) $$\bar{H}=\left\{\bar{\eta }(\cdot )|(\bar{U}(\cdot ),\bar{\chi }(\cdot ))\in \bar{U}\right\},$$

where $\bar{\eta }\left(\cdot \right)$ denotes a kinematic simulation procedure that given SOG and course trajectories, Ū(·) and $\bar{\chi }\left(\cdot \right)$, in ${\bar{U}}_{d}$ computes the vessel pose. See Eriksen and Breivik (2019) and Eriksen et al. (2019) for more details on the trajectory generation procedure.

In order to converge toward the trajectory specified by the mid-level algorithm, a desired acceleration is computed based on a line-of-sight guidance scheme. In Eriksen and Breivik (2019) and Eriksen et al. (2019), the samples closest to the desired acceleration in (35) are replaced with the desired acceleration, given that this is vessel-feasible. A problem with this, is that when operating at high speeds, the possible acceleration may not be symmetric, resulting in that zero acceleration (hence keeping a constant speed and course), may not be part of the search space. This can cause undesirable behavior, since the BC-MPC algorithm will be unable to keep the speed and course constant, which can cause oscillatory behavior. In this paper, we therefore propose to move the acceleration samples closest to zero, and adding the desired acceleration as a separate sample, given that it is vessel feasible. This will make sure that keeping a constant speed and course, as well as a trajectory converging toward the desired trajectory is included in the search space.

Given the predicted trajectories, the algorithm finds the optimal desired SOG and course trajectory for the vessel controller ${\text{u}}_{d}^{*}\left(\cdot \right)=\left[U_{d}{\left(\cdot \right)}^{*},\chi_{d}{\left(\cdot \right)}^{*}\right]$ as

(37) $${\text{u}}_{d}^{*}(\cdot )=\underset{\left({\bar{\eta }}_{k}(\cdot ),{\text{u}}_{d,k}(\cdot ))\in (\bar{ℋ},U_{d}\right)}{\text{argmin}}G({\bar{\eta }}_{k}(\cdot ),{\text{u}}_{d,k}(\cdot );{\text{p}}_{d}^{\text{mid}}(\cdot )),$$

where the objective function is given as

(38) $$\begin{array}{l} G(\bar{\eta }(\cdot ),{\text{u}}_{d}(\cdot );{\text{p}}_{d}^{\text{mid}}(\cdot ))=w_{\text{al}}\text{align}(\bar{\eta }(\cdot );{\text{p}}_{d}^{\text{mid}}(\cdot ))\\ \;+w_{\text{av},\text{m}}{\text{avoid}}_{\text{m}}(\bar{\eta }(\cdot ))+w_{\text{av,s}}{\text{avoid}}_{\text{s}}(\bar{\eta }(\cdot ))\\ \;+w_{\text{t},U}{\text{tran}}_{U}({\text{u}}_{d}(\cdot ))+w_{\text{t},\chi }{\text{tran}}_{\chi }({\text{u}}_{d}(\cdot )). \end{array}$$

The variables *w*_al_, *w*_av, m_, *w*_av, s_, *w*_t, *U*_, *w*_t, χ_ > 0 are tuning parameters, while $\text{align}\left(\bar{\eta }\left(\cdot \right);{\text{p}}_{d}^{\text{mid}}\left(\cdot \right)\right)$ measures the alignment between a candidate trajectory $\bar{\eta }\left(\cdot \right)$ and the desired trajectory from the mid-level algorithm ${\text{p}}_{d}^{\text{mid}}\left(\cdot \right)$. The function ${\text{avoid}}_{\text{m}}\left(\bar{\eta }\left(\cdot \right)\right)$ ensures COLAV of moving obstacles by penalizing trajectories close to obstacles, using a non-symmetric obstacle ship domain designed with the COLREGs in mind. The function ${\text{avoid}}_{\text{s}}\left(\bar{\eta }\left(\cdot \right)\right)$ ensures COLAV of static obstacles by introducing an occupancy grid, while tran_*U*_(***u***_*d*_(·)) and tran_χ_(***u***_*d*_(·)) introduces transitional costs to avoid shattering. The transitional terms penalize deviations from the planned trajectory of the previous iteration, unless changing to the trajectory corresponding by the desired acceleration. See Eriksen and Breivik (2019) and Eriksen et al. (2019) for more details and descriptions of the terms.

## 6. Simulation Results

The hybrid COLAV system is verified through simulations, which are present in this section. The simulations include ocean current and both static and moving obstacles. We include moving obstacles both acting in compliance with the COLREGs, and violating the COLREGs.

### 6.1. Simulation Setup

The simulations are performed in MATLAB on a computer with an 2.8 GHz Intel Core i7 processor running macOS Mojave, using CasADi (Andersson et al., 2019) and IPOPT (Wächter and Biegler, 2005) for implementing the high-level and mid-level algorithms. The simulator is built upon the mathematical model of the Telemetron ASV described in section 2, and the model-based speed and course controller in Eriksen and Breivik (2018) is used as the vessel controller.

The parameters of the high-level algorithm are listed in Table 1. The number of prediction steps *N*_hi_ is chosen to achieve a time step length *h* = *t*_max_/*N*_hi_ < 1.5 s, which seems to be a good compromise between capturing the relevant system dynamics and having a feasible computational requirement.

**Table 1:** Tuning parameters for the high-level algorithm.

**Param**.	**Value**	**Comment**
*t*_max_		Maximum trajectory time
Scenario 1	1420 s	
Scenario 2	1420 s	
Scenario 3	725 s	
*N*_hi_	1000	Number of prediction steps
*K*_*e*_	1.0 s^3^/m	Energy penalty gain
*K*_δ_	1.0	Quadratic yaw control penalty gain
*L*_*m*_	4.0 m	Length between control origin and outboard motor

The mid-level algorithm is implemented using the parameters in Table 2.

**Table 2:** Tuning parameters for the mid-level algorithm.

**Param**.	**Value**	**Comment**
${\bar{d}}_{\text{CPA}}^{\text{enter}}$	900 m	State machine *d*_CPA_ entry criteria
$\;{\underline{d}}_{\text{CPA}}^{\text{exit}}$	2000 m	State machine *d*_CPA_ exit criteria
[$\;{\underline{t}}_{\text{CPA}}^{\text{enter}},{\bar{t}}_{\text{CPA}}^{\text{exit}}$]	[0, 270] s	State machine *t*_CPA_ entry criteria
[$\;{\underline{t}}_{\text{CPA}}^{\text{exit}},\;{\bar{t}}_{\text{CPA}}^{\text{exit}}$]	[-20, 290] s	State machine *t*_CPA_ exit criteria
${\bar{t}}_{\text{crit}}^{\text{EM,enter}}$	20 s	Emergency state *t*_crit_ entry criteria
${\underline{t}}_{\text{crit}}^{\text{EM},\text{exit}}$	25 s	Emergency state *t*_crit_ exit criteria
*h*	10 s	Step size
*N*_*p*_	36	Number of prediction steps
*K*_*p*_	0.02	Position error scaling
σ	1	Huber loss function threshold
$K_{\overset{∙}{U}}$	0.3	SOG-derivative penalty term scaling
$K_{\overset{∙}{\chi }}$	2.5	Course-derivative penalty term scaling
*K*_HO_	40	Head-on potential function scaling
[α_*x*, HO_, α_*y*, HO_]	[1/500, 1/400]	Head-on potential function steepness parameters
*x*_0, HO_	1000 m	Head-on potential function attenuation parameter
*K*_GW_	40	Give-way potential function scaling
[α_*x*, GW_, α_*y*, GW_]	[1/400, 1/500]	Give-way potential function steepness parameters
*y*_0, GW_	−500 m	Give-way potential function attenuation parameter
*K*_SO_	3	Stand-on function scaling
*K*_EM_	3	Emergency function scaling
*K*_ξ_	[0.1, 1, 10, 100, ∞]	Iterative slack variable cost
*x*_*a*_	600 m	Moving obstacle ellipsis major axis size
*y*_*a*_	225 m	Moving obstacle ellipsis minor axis size

The slack variable cost *K*_ξ_ has five elements, implying that we use five steps in our homotopy scheme. The mid-level NLP is initially warm started with the solution from the previous iteration, while each step in the homotopy scheme is warm started with the solution from the previous step of the homotopy scheme, converging toward the solution without slack on the constraints. To reduce the computational load and increase the predictability of the mid-level algorithm, we utilize six steps of each planned mid-level trajectory, only running the mid-level algorithm every 60 s. This implies that six steps of the predicted solution will be implemented before computing a new solution, which further implies that the state machine is also only run every 60 s. If the mid-level algorithm fails in finding a feasible solution, the algorithm will re-use the solution from the last iteration. This may for instance happen if the algorithm tries to compute a solution while being inside a moving obstacle ellipse, which sometimes can be the case when an obstacle is exiting an emergency or stand-on state. The BC-MPC algorithm is run every 5 s, with parameters as described in Eriksen and Breivik (2019). An update rate of 5 s is considered sufficient due the typically large maneuvering margins at sea. It is also worth noting that the detection and tracking system can represent a significant time delay, especially for radar-based systems (Eriksen et al., 2019). For confined and congested areas the BC-MPC algorithm may need to be run at a higher rate, which also imposes requirements for high-bandwidth obstacle estimates. Static obstacles are padded with a safety margin of 150 m for the high-level and mid-level algorithms, while the BC-MPC algorithm uses a safety margin of 100 m for static obstacles. The reason for having a smaller static obstacle safety margin for the BC-MPC algorithm is that it tends to struggle with following trajectories on the static obstacle boundaries. The BC-MPC algorithm would hence not be able to follow the nominal trajectory if the static obstacle safety margin was the same as for the mid-level and high-level algorithms.

The simulations are performed without any noise on the obstacle estimates, providing the algorithms with exact information about the obstacles position, course, and speed. The BC-MPC algorithm has previously been shown to perform well with noisy and uncertain obstacle estimates in full-scale experiments using radar-based detection and tracking of obstacles (Eriksen and Breivik, 2019; Eriksen et al., 2019). The mid-level algorithm is likely to have a larger requirement to low noise levels on the obstacle estimates, since the state machine in the mid-level algorithm depends on logic and discrete switching. However, the algorithm is also run less frequently, reducing the required bandwidth of the obstacle estimates, possibly allowing using smoothing or tracking filters with a lower process noise if necessary. It may also be feasible to make the mid-level algorithm depend on data from the automatic identification system, which typically have much lower noise levels than radar-based tracking systems, while being subject to robustness issues (Harati-Mokhtari et al., 2007).

We present three scenarios, which demonstrate different important properties of the hybrid COLAV system:

**Table:** 

**Scenario 1**	This scenario contains two static obstacles, and four moving obstacles of which all comply with the COLREGs. The moving obstacles demonstrate stand-on, give-way and head-on situations.
**Scenario 2**	This scenario contains one static and five moving obstacles. The moving obstacles demonstrate stand-on with an obstacle ignoring the COLREGs, an overtaking and a simultaneous head-on, give-way and stand-on situation with obstacles complying with the COLREGs.
**Scenario 3**	This scenario contains two moving obstacles, which suddenly perform dangerous maneuvers close to the ownship, displaying the use of the emergency state.

### 6.2. Scenario 1

**Figure 7:**
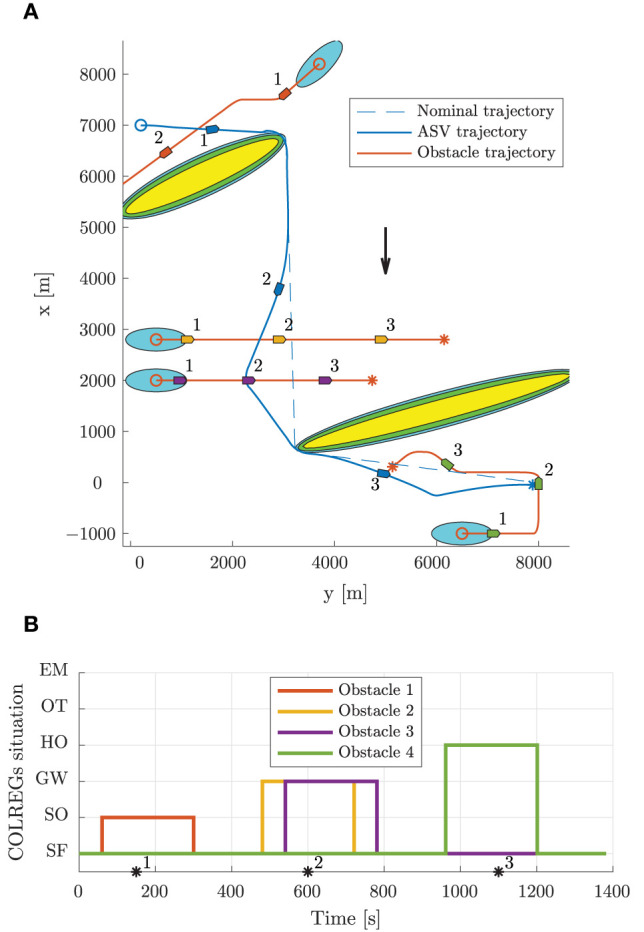
Scenario 1: trajectory and COLREGs interpretation. The text marks denote the time steps [150, 600, 1100] s. **(A)** Trajectory plot. The initial position of the ownship and obstacles are shown with circles, with the blue ellipses illustrating the moving obstacle ellipse size. The vessel patches, which are overexaggerated for visualization, mark the ownship and obstacle poses at given time stamps. The static obstacles are shown in yellow, with the BC-MPC and mid-level safety margins enclosed around. The black arrow indicates the ocean current direction. **(B)** Output from the state machine for each obstacle. The asterisks mark time stamps, and the colors correspond to the obstacle patch colors in the trajectory plot.

Scenario 1 contains two static obstacles, four moving obstacles, an ocean current of [−2, 0]^⊤^ m/s and is shown in Figure 7. The high-level planner plans a nominal trajectory between the initial and goal positions at [7000, 200]^⊤^ m and [0, 7900]^⊤^ m, respectively. The first obstacle is in a stand-on situation, where it is required to maneuver in order to avoid collision with the ownship, which is required to stand on. As shown in Figure 7B, the first obstacle is quickly considered as a stand-on situation, at which the mid-level algorithm disregards the obstacle and continues with the current speed and course. Following this, the obstacle maneuvers in accordance to the COLREGs, and we avoid collision. After the first static obstacle, we encounter two crossing vessels where the ownship is deemed the give-way vessel. In accordance with the COLREGs, we maneuver to starboard in order to pass behind both obstacles. Notice that the second give-way obstacle is detected as a give-way situation later than the first, since the entry criteria in the state machine includes the time to CPA, which is higher for the second give-way obstacle. After avoiding the two give-way obstacles, we converge toward the nominal trajectory and encounter a head-on situation. This is correctly identified by the state machine as head on, and we maneuver to starboard in order to avoid collision. Notice that even though the obstacle maneuvers, we keep the obstacle in the head-on state until we have passed it.

**Figure 8:**
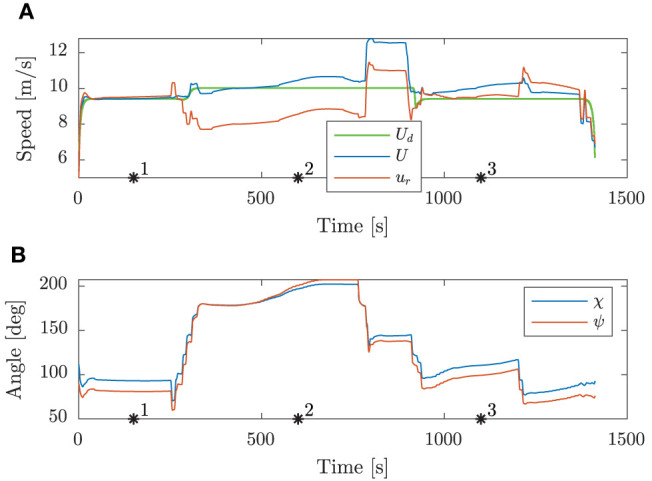
Scenario 1: speed and angular trajectories. The asterisks mark the same time samples as in Figure 7. **(A)** Speed trajectories. **(B)** Angular trajectories.

Figure 8 shows the speed and angular trajectories during Scenario 1, where the desired speed is calculated as the nominal speed at the closest point on the nominal trajectory given the ownship position. From this, we see that the mid-level and BC-MPC algorithms manage to track the desired nominal speed before and after the first static obstacle, where no obstacles require maneuvering away from the nominal trajectory. Notice that when encountering the two crossing obstacles, the mid-level algorithm chooses to slowly change the course, which is due to the attenuation of the give-way potential function and the large distance between the vessels. It would be better to make a clear course change, which is a subject of tuning. After passing the two crossing obstacles, the mid-level algorithm increases the speed in order to get back to the nominal trajectory, which is due to the algorithm attempting to keep the speed projected on the nominal trajectory equal as the desired nominal speed. Furthermore, notice that the mid-level algorithm actively controls the relative surge speed in order achieve the desired SOG, which is clearly seen when passing the first static obstacle.

### 6.3. Scenario 2

**Figure 9:**
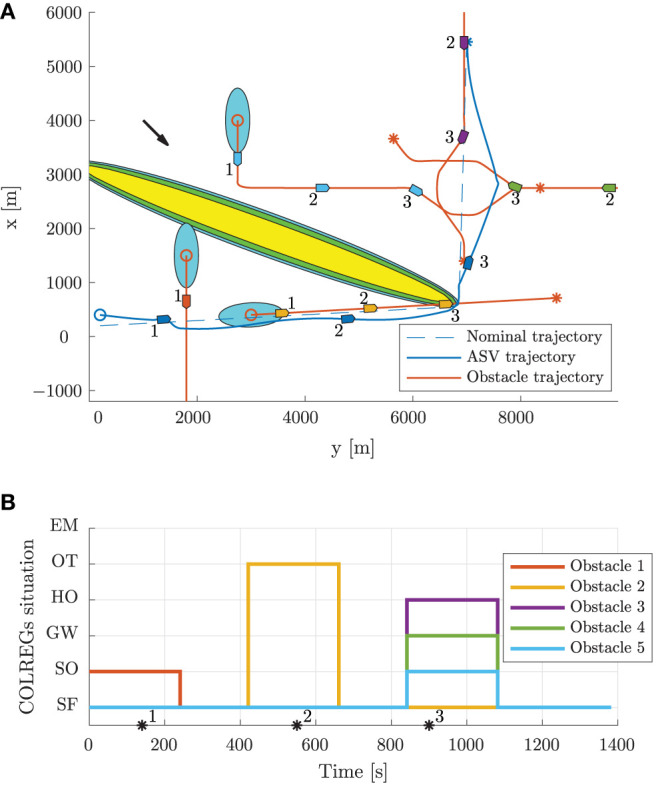
Scenario 2: trajectory and COLREGs interpretation. The text marks denote the time steps [140, 550, 900] s. **(A)** Trajectory plot. The initial position of the ownship and obstacles are shown with circles, with the blue ellipses illustrating the moving obstacle ellipse size. The vessel patches, which are overexaggerated for visualization, mark the ownship and obstacle poses at given time stamps. The static obstacles are shown in yellow, with the BC-MPC and mid-level safety margins enclosed around. The black arrow indicates the ocean current direction. **(B)** Output from the state machine for each obstacle. The asterisks mark time stamps, and the colors correspond to the obstacle patch colors in the trajectory plot.

Scenario 2, shown in Figure 9, is more complex than Scenario 1, with a total of five moving obstacles, and has an ocean current of [−1, 1]^⊤^ m/s. The high-level planner plans a nominal trajectory between the initial and goal positions at [200, 200]^⊤^ m and [5500, 7000]^⊤^ m, respectively. The first obstacle is a crossing vessel, which similarly as in Scenario 1 is deemed to give way for the ownship, which should keep the current speed and course. However, in this scenario, the obstacle violates the COLREGs by not maneuvering in order to avoid collision. Therefore, the BC-MPC algorithm maneuvers to avoid collision when the obstacle gets so close that the safety margins of the BC-MPC algorithms is violated. The BC-MPC algorithm maneuvers to port, as advised by COLREGs Rule 17 for crossing situations where the stand-on vessel has to maneuver, and safely avoid the first obstacle. The second obstacle is overtaken by the ownship, and correctly considered as an overtaking situation by the state machine. For such an situation, there is no requirement on how the ownship should maneuver, except keeping clear from the overtaken vessel. After passing the second obstacle, we encounter a complex situation with simultaneous head-on, give-way and stand-on obligations. In this situation, each vessel, including the ownship, finds itself in a situation where a head-on and a give-way situation require starboard maneuvers, while a stand-on situation requires the vessel to keep the current speed and course. However, head-on and give-way obligations should be prioritized higher than stand-on situations, and the situation is quite easily solved by each vessel maneuvering to starboard and passing behind the vessel crossing from starboard. The mid-level algorithm solves this situation with the desirable behavior, and converges toward the nominal trajectory after the situation is resolved. As shown in Figure 9B, the state machine interprets the situations correctly.

**Figure 10:**
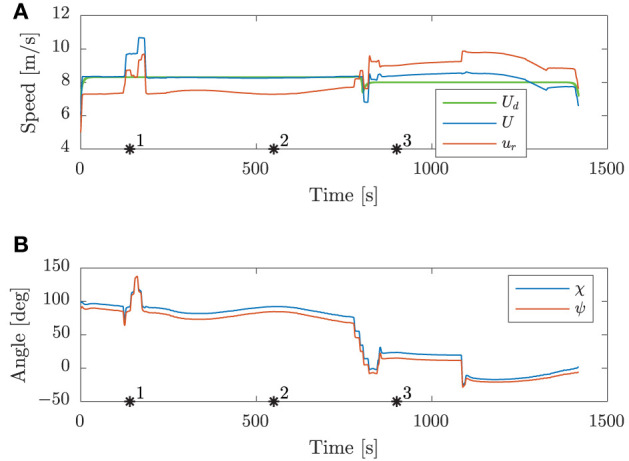
Scenario 2: speed and angular trajectories. The asterisks mark the same time samples as in Figure 9. **(A)** Speed trajectories. **(B)** Angular trajectories.

From the speed trajectory in Figure 10 it is clear that the mid-level algorithm follows the desired nominal speed also when overtaking the second obstacle.

### 6.4. Scenario 3

**Figure 11:**
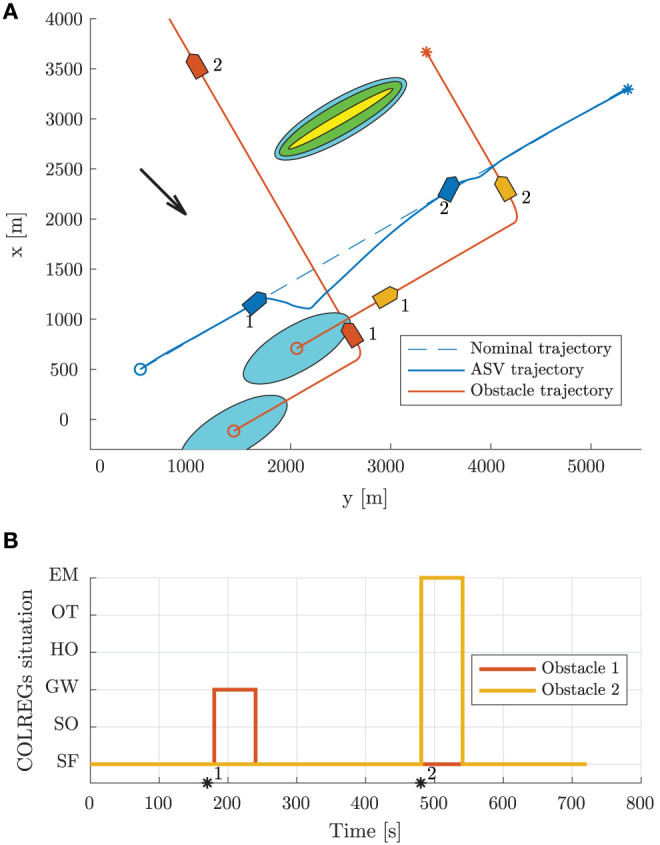
Scenario 3: trajectory and COLREGs interpretation. The text marks denote the time steps [170, 480] s. **(A)** Trajectory plot. The initial position of the ownship and obstacles are shown with circles, with the blue ellipses illustrating the moving obstacle ellipse size. The vessel patches, which are overexaggerated for visualization, mark the ownship and obstacle poses at given time stamps. The static obstacles are shown in yellow, with the BC-MPC and mid-level safety margins enclosed around. The black arrow indicates the ocean current direction. **(B)** Output from the state machine for each obstacle. The asterisks mark time stamps, and the colors correspond to the obstacle patch colors in the trajectory plot.

Scenario 3, shown in Figure 11, contains two moving obstacles on parallel courses with the ownship, and has an ocean current of [−1, 1]^⊤^ m/s. The high-level planner plans a nominal trajectory between the initial and goal positions at [500, 500]^⊤^ m and [3328, 5399]^⊤^ m, respectively, which results in a straight line trajectory with a course angle of 60°. The first obstacle travels at a higher speed than the ownship, while the second one travels at a lower speed and will be overtaken by the ownship. Since the obstacles are on parallel paths with the obstacle, the time to CPA is sufficiently high such that the obstacles are in the safe state, even though the vessels are quite close. However, both obstacles make sudden maneuvers to port dangerously close to the ownship and enters on a crossing course with the ownship. With respect to the COLREGs, the ownship is required to give way to both obstacles since they are crossing from the ownship's starboard side. One can, however, argue that the maneuvers displayed by the obstacles are dangerous and displays poor seamanship, such that the ownship should not be held accountable if a collision occurred. Nevertheless, the hybrid COLAV system manages to avoid both obstacles. As seen in Figure 11B, the first obstacle is sufficiently far away from the ownship to be considered as a give-way situation when the state machine interprets the situation, and the mid-level algorithm plans a trajectory passing behind the first obstacle. The second obstacle maneuvers to port even closer to the ownship, resulting in the distance to the critical point being within the threshold for entering the emergency situation when the state machine interprets the situation. In this situation, the mid-level algorithm disregards the obstacle and leaves it to the BC-MPC algorithm to avoid collision.

**Figure 12:**
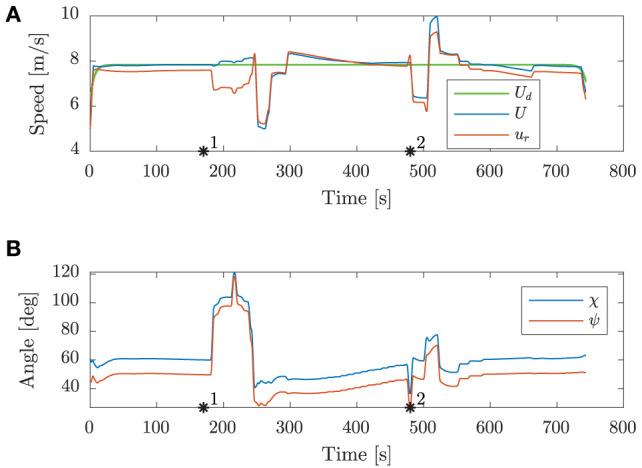
Scenario 3: speed and angular trajectories. The asterisks mark the same time samples as in Figure 11. **(A)** Speed trajectories. **(B)** Angular trajectories.

As seen in Figure 12, the mid-level algorithm both reduces the speed and changes the course to avoid the first obstacle. When approaching the second obstacle, the BC-MPC algorithm initiates a speed reduction, and after some time also maneuver to starboard in order to pass behind the obstacle and resolve the situation.

### 6.5. Simulation Summary

The simulation results show that the hybrid COLAV system is able to handle a wide range of situations, while also behaving in an energy-optimal way when moving obstacles are not interfering with the ownship trajectory. Table 3 shows the minimum distance to static and moving obstacles for the scenarios.

**Table 3:** Minimum distance to static and moving obstacles for the simulation scenarios.

**Scenario**	**Minimum distance to static obstacles (m)**	**Minimum distance to moving obstacle number (m)**				
		**1**	**2**	**3**	**4**	**5**
Scenario 1	93.7	634.3	596.3	522.7	726.8	–
Scenario 2	118.2	185.5	228.3	1,097.2	575.6	842.3
Scenario 3	1123.8	326.4	106.6	–	–	–

The minimum distance to static obstacles is in Scenario 1 below the safety region size of the BC-MPC algorithm, which is intentional and caused by the algorithm using a smooth penalty function for interpreting static obstacles. The penalty function value increases linearly when moving further into the safety region, see Eriksen and Breivik (2019) for more details. The minimum distance to moving obstacles is a bit difficult to interpret, since the obstacle ship domains are non-circular, implying that the required clearance depends on relative position of the ownship with respect to the moving obstacles. However, we see that we have a larger clearance in head-on, give-way and stand-on situations where the obstacles comply with the COLREGs, and do not perform dangerous maneuvers (as in Scenario 3), compared to overtaking situations. The reason for this is that when overtaking (obstacle 2 in Scenario 2), we pass the obstacle on a parallel course, resulting in the minor axis of the moving obstacle ellipsis indicating the required clearance. Furthermore, we see that obstacle 1 in Scenario 2, which ignores its give-way obligation, comes significantly closer than other crossing obstacles except for those in Scenario 3. The reason for this is that the BC-MPC algorithm, which handles this situation, has a lower clearance requirement than the mid-level algorithm, which still should be considered as safe. In Scenario 3, the two obstacles display poor seamanship, and behave dangerously. Obstacle 1 is handled by the mid-level algorithm and passed with a clearance lower than the major axis of the mid-level algorithm, which is caused by the BC-MPC algorithm “cutting the corner.” The clearance should still be considered safe since we are behind the obstacle, and the clearance requirements of the BC-MPC algorithm is enforced. Obstacle 2, which is placed in the emergency state and handled by the BC-MPC algorithm, is passed with a clearance of only 106.6 m. This is lower than the clearance to Obstacle 1 in Scenario 2 (which violated its stand-on requirement), and is due to the BC-MPC algorithm having a non-symmetric obstacle ship domain function allowing for a smaller clearance when passing behind an obstacle than in front.

For the three scenarios, the high-level planner used an average of 67 s with a maximum of 93 s to compute the solution. Since the high-level planner is intended to be run off-line, this is well within reasonable limits. The mid-level algorithm used 0.60 s on average, and a maximum of 2.1 s, which we consider to be computationally feasible since the mid-level algorithm only is run every 60 s. The BC-MPC algorithm used 0.29 s on average, and a maximum of 0.63 s, which we also consider to be real-time feasible when the BC-MPC algorithm is run every 5 s. The BC-MPC algorithm is highly parallelizable, which could reduce the BC-MPC runtime by a large magnitude if required. The mid- and high-level algorithms may not return solutions as they are non-convex optimization problems, but the BC-MPC algorithm makes the hybrid COLAV system real-time feasible since it always will find a (potentially sub-optimal) solution.

## 7. Conclusion

In this paper, we have presented a three-layered hybrid COLAV system, compliant with COLREGs rules 8 and 13–17. As part of this, we have further developed the MPC-based mid-level COLAV algorithm in Eriksen and Breivik (2017b) and Bitar et al. (2019a) to comply with COLREGs rules 13–16 and parts of Rule 17, which includes developing a state machine for COLREGs interpretation. The hybrid COLAV system has a well-defined division of labor, including an inherent understanding of COLREGs Rule 17, where the mid-level algorithm obeys stand-on situations, while the BC-MPC algorithm handles situations where give-way vessels do not maneuver.

The hybrid COLAV system is verified through simulations, where we in three scenarios challenge the system with a number of different situations. The scenarios include multi-obstacle situations with multiple simultaneously active COLREGs rules, and situations where obstacles violate the COLREGs. Collision is avoided in all the scenarios, and we show that the ownship follows an energy-optimized trajectory generated by the high-level planner when moving obstacles do not interfere with this trajectory.

For further work, we suggest to:

- Investigate if using situation-dependent entry and exit criteria parameters in the state machine improves the performance.
- Expand the state machine with the possibility of transitioning from head-on, give-way and overtaking states to the emergency state for situations where obstacles behave dangerously or hostile.
- Develop a methodology for deciding tuning parameters.
- Perform simulations with noisy obstacle estimates to investigate how the state machine and mid-level algorithm respond to this.
- Explore the possibilities for integrating the COLREGs interpretation in the mid-level NLP, relaxing the assumption of the current COLREGs situation being valid for the entire prediction horizon.
- Investigate the possibility of including static obstacles from e.g., ENCs in the high- and mid-level algorithms.
- Simulate scenarios where multiple vessels running the hybrid COLAV system interact with each other.
- Validate the hybrid COLAV system in full-scale experiments.

## Data Availability Statement

The datasets generated for this study are available on request to the corresponding author.

## Author Contributions

The work in this article was the result of a collaboration between B-OE and GB, supervised by MB and AL. The contributions to the mid-level and short-term algorithms are made by B-OE, while the COLREGs interpreter was developed in collaboration between GB and B-OE. GB has implemented the high-level planner, and prepared this for integration with the developed simulator. B-OE has taken lead on writing the paper, in collaboration with GB. MB and AL have provided valuable feedback in the writing process.

### Conflict of Interest

The authors declare that the research was conducted in the absence of any commercial or financial relationships that could be construed as a potential conflict of interest.

## Footnotes

^1^https://www.wsj.com/articles/norway-takes-lead-in-race-to-build-autonomous-cargo-ships-1500721202 (accessed May 22, 2019).

^2^https://www.marinemec.com/news/view,rollsroyce-and-finferries-demonstrate-worlds-first-fully-autonomous-ferry_56102.htm (accessed April 11, 2019).

^3^Rule 17 *allows* the stand-on vessel to maneuver when it becomes apparent that the give-way vessel does maneuver to avoid collision. If the vessels are so close that the give-way vessel cannot avoid collision by itself, Rule 17 *requires* the stand-on vessel to maneuver.

^4^http://www.emodnet.eu/ (accessed December 11, 2019).
